# Reservoir differences across HIV-1 subtypes: a systematic review to guide cure research

**DOI:** 10.3389/fimmu.2026.1870118

**Published:** 2026-07-29

**Authors:** Myrthe S. Willemsen, Anniek A. N. Tanja, Sody Munsaka, Damalie Nakanjako, Thumbi Ndung’u, Maria A. Papathanasopoulos, Kevin Jenniskens, Giulia Sinatti, Jori Symons, Annemarie M. J. Wensing, Monique Nijhuis, Ganna Rozhnova

**Affiliations:** 1 Julius Center for Health Sciences and Primary Care, University Medical Center Utrecht, Utrecht University, Utrecht, Netherlands; 2 Center for Complex Systems Studies (CCSS), Utrecht University, Utrecht, Netherlands; 3 Translational Virology, Department of Medical Microbiology, University Medical Center Utrecht, Utrecht, Netherlands; 4 Department of Biomedical Sciences, School of Health Sciences, University of Zambia, Lusaka, Zambia; 5 Department of Medicine, School of Medicine, Makerere University College of Health Sciences, Kampala, Uganda; 6 Infectious Diseases Institute, Makerere University, Kampala, Uganda; 7 Africa Health Research Institute, Durban, South Africa; 8 HIV Pathogenesis Programme, The Doris Duke Medical Research Institute, University of KwaZulu-Natal, Durban, South Africa; 9 Ragon Institute of Mass General Brigham (MGB), Massachusetts Institute of Technology (MIT) and Harvard, Cambridge, MA, United States; 10 Institute of Infection, Immunity and Transplantation, University College London, London, United Kingdom; 11 HIV Pathogenesis Research Unit, Department of Molecular Medicine and Haematology, Faculty of Health Sciences, University of Witswatersrand, Johannesburg, South Africa; 12 Cochrane Netherlands, University Medical Center Utrecht, Utrecht University, Utrecht, Netherlands; 13 Department of Social and Cultural Anthropology, Vrije Universiteit Amsterdam, Amsterdam, Netherlands; 14 Amsterdam Institute of Global Health and Development (AIGHD), Amsterdam, Netherlands; 15 Translational Virology, Department of Global Public Health and Bioethics, University Medical Center Utrecht, Utrecht, Netherlands; 16 BioISI—Biosystems and Integrative Sciences Institute, Faculty of Sciences, University of Lisbon, Lisbon, Portugal; 17 Faculty of Sciences, University of Lisbon, Lisbon, Portugal

**Keywords:** HIV cure, HIV-1, humans, subtype, viral latency, viral reservoir

## Abstract

**Introduction:**

Despite effective antiretroviral therapy, HIV-1 persists as a viral reservoir and remains a major barrier to a durable cure. Reservoir size and dynamics are central determinants of the success of cure strategies. Efforts to characterize and target the HIV-1 reservoir have primarily focused on people with HIV-1 subtype B in Western Europe and North America. This emphasis has limited our understanding of reservoir size and dynamics in individuals with non-B subtypes, which account for most infections globally, particularly in high-burden regions such as sub-Saharan Africa. This systematic review synthesizes evidence on HIV-1 reservoir size in individuals with non-B subtypes and compiles reported reservoir outcomes alongside demographic and clinical metadata.

**Methods:**

PubMed and EMBASE were searched for studies quantifying the HIV-1 reservoir in peripheral blood from individuals with non-B subtypes. Eligible studies quantified reservoir size in at least three non-B samples stratified by subtype or directly compared measurements between subtypes. Data were extracted on subtype, reservoir size, statistical comparisons thereof, and key demographic and clinical data. Study quality was assessed using a pre-defined, custom set of criteria. Data were synthesized by organizing within-study statistical comparisons by subtype in a schematic overview to indicate the direction of effect.

**Results:**

Of 1589 unique records, 40 studies quantifying HIV-1 reservoir size in individuals with non-B subtypes were included. Records excluded at full-text screening commonly lacked viral subtype determination and/or subtype-stratified outcomes. Comparisons between subtype B and pooled non-B subtypes suggest a larger reservoir for subtype B. Substantial differences in study design, (meta)data reporting, and comparability limited direct comparison across studies.

**Conclusion:**

Strengthening knowledge on the HIV-1 subtype-specific reservoir is essential for the development of effective and globally relevant cure strategies. The available data point toward possible differences in reservoir size between subtype B and non-B subtypes. To improve further assessment, we developed recommendations urging studies to use standardized subtyping methods, report viral subtype according to Los Alamos guidelines, and provide detailed individual-level outcomes and metadata, including disease stage at treatment initiation, ART duration, and viral suppression status. Furthermore, we strongly recommend using multi-subtype reservoir assays that are cross-validated to enhance comparability across studies.

**Systematic Review Registration:**

https://www.crd.york.ac.uk/PROSPERO/view/CRD420251074799, identifier CRD420251074799.

## 1 Introduction

Human immunodeficiency virus type 1 (HIV-1) is characterized by substantial genetic diversity and is classified into multiple subtypes that are unevenly distributed worldwide. Subtype C, responsible for nearly half of global infections, predominates in southern Africa and India, whereas subtype B accounts for 11% of infections worldwide and is largely confined to Europe, Australia, and the Americas (1). Although current combinatorial antiretroviral therapy (ART) can effectively suppress viral replication, it does not eliminate the virus, necessitating lifelong treatment (2, 3). Efforts to achieve an HIV-1 cure have rapidly advanced over the past decades, giving new insights into viral persistence and strategies to achieve ART-free viral control. However, these advances have been largely driven by research in high-income settings, where subtype B predominates, while non-B subtypes remain underrepresented in HIV-1 cure research. Viral subtype diversity can influence reservoir size, composition, and dynamics, which are key determinants of responses to curative interventions (4–6). The underrepresentation of non-B HIV-1 subtypes in reservoir and cure studies may therefore limit the generalizability of current findings. As cure strategies advance toward clinical translation, investigating subtype-specific differences in the viral reservoir is essential to ensure that these approaches are effective for populations most affected by HIV-1.

The primary barrier to an HIV-1 cure is the latent viral reservoir, which consists of HIV-1-infected cells, predominantly CD4+ T cells, harboring integrated, transcriptionally silent proviruses (3, 7). These proviruses can persist for decades under suppressive ART and retain the capacity to reactivate and produce infectious viral particles upon cellular stimulation, leading to viral rebound when ART is interrupted (8–10). Consequently, most HIV-1 cure strategies aim to eliminate or permanently control this reservoir, for example, by inducing viral expression followed by immune-mediated clearance (shock and kill), transcriptional silencing (block and lock), or reducing the pool of rebound-competent HIV-1-infected cells through immunotherapeutic or gene-based approaches (11, 12).

Only a small fraction of the HIV-1 reservoir contributes to viral rebound, as most integrated proviruses are defective and only a minority are intact and capable of producing infectious virus upon reactivation (5, 13) (see **Table 1** for definitions and quantification methods of these distinct reservoir components). The replication-competent fraction is considered the most clinically relevant component of the reservoir and a major determinant of the success of curative strategies (5). This highlights the importance of distinguishing between defective and replication-competent proviruses when assessing the reservoir. However, quantification of the replication-competent reservoir is labor-intensive and costly, limiting its widespread use. Simpler and more scalable measures, such as total and intact HIV-1 DNA have demonstrated associations with clinically relevant outcomes, including time to viral rebound and response to curative interventions (14).

**Table 1:** Definition of the HIV-1 reservoir component and quantification across the 40 studies included in the systematic review.

HIV-1 quantification	Definition	Common assays	Typical units	Number of studies
Total HIV-1 DNA	Integrated and non-integrated cell-associated HIV-1 DNA	qPCR or dPCR for total HIV-1 DNA	Copies per million cells	35
Integrated HIV-1 DNA	Stably integrated HIV-1 DNA in the host genome	Alu–PCR–based assays, linker-mediated PCR	Copies per million cells	5
Intact HIV-1 DNA	HIV-1 proviruses without major genetic defects and potentially replication competent	Intact Proviral DNA Assay (IPDA) or near full-length individual proviral sequencing (FLIPS)	Copies per million cells	11
Inducible HIV-1	HIV-1 proviruses that can be transcriptionally reactivated to produce viral RNA or virions upon stimulation	Inducible RNA assays	Infectious units per million cells (IUPM)	5
Replication-competent HIV-1	HIV-1 proviruses capable of producing infectious virus	Quantitative viral outgrowth assay (QVOA)	IUPM	4

Importantly, the HIV-1 reservoir is not static but shaped by multiple determinants. Treatment-related factors, such as treatment initiation, duration, and regimen, play an important role: initiation of ART during acute infection is associated with a smaller viral reservoir compared with initiation during chronic HIV-1 infection (6, 15, 16). During ART, total and intact HIV-1 DNA decline, with the most pronounced decrease occurring within the first year of treatment, after which the size of the defective proviral reservoir plateaus (6, 17–20). Beyond treatment-related factors, host and environmental factors such as biological sex, ethnicity, and co-infections are thought to additionally influence HIV-1 reservoir size (6, 21–23). Finally, among viral factors, HIV-1 subtype may be an important contributor to variation in reservoir size, dynamics, and composition (6, 24, 25).

HIV-1 subtypes are genetically highly diverse, and this diversity has been associated with phenotypic differences, such as viral fitness, co-receptor usage, and integration site selection (26–31). Clinical differences in immune recovery, antiretroviral drug resistance development, and viral rebound have also been observed between subtypes and may reflect subtype-specific differences in the viral reservoir (24, 32–36). Supporting this, subtype-specific variation in the long terminal repeat (LTR), which regulates viral transcriptional activity, and differences in responsiveness to latency reversal agents (LRAs) suggest that subtype may affect reservoir size and cure-related outcomes (29, 37, 38). Despite these indications, the extent to which HIV-1 subtype independently contributes to reservoir heterogeneity remains unclear. To address this knowledge gap, we performed a systematic review to synthesize available evidence on HIV-1 reservoir size in peripheral blood across different HIV-1 subtypes and compile reported reservoir outcomes alongside demographic and clinical data.

## 2 Materials and methods

### 2.1 Search strategy

The systematic review was conducted in accordance with the Preferred Reporting Items for Systematic Reviews and Meta-Analyses (PRISMA) guidelines (39, 40). The PRISMA checklist is available in **Supplementary Table 1**. The review protocol was registered in the International Prospective Register of Systematic Reviews (PROSPERO; record ID CRD420251074799). A literature search was performed in EMBASE and MEDLINE, through PubMed on July 3, 2025, without restriction on publication date. Search terms were developed in consultation with an information specialist and combined terms related to HIV-1, viral subtype, and the HIV-1 reservoir. The full search string for each database can be found in **Supplementary Appendix 1**. In addition, the reference lists of all included studies and relevant review articles were manually screened to identify potentially eligible studies not captured by the electronic searches.

### 2.2 Study selection

All titles and abstracts from the database searches were imported into Rayyan software (41). Duplicate records were identified using a two-step process: (1) automated deduplication based on exact title match, disregarding cases and punctuation, and (2) manual review of records based on similarity. The remaining records were independently screened for eligibility by two authors (MSW and AANT) at both title-abstract and full-text screening stages. We only included peer-reviewed research articles. Articles were included if they quantified the size of the HIV-1 reservoir, including total HIV-1 DNA, integrated HIV-1 DNA, intact HIV-1 DNA, inducible HIV-1, and replication-competent HIV-1, in peripheral blood samples from at least three individuals with non-B subtypes on or off ART. Note that throughout this review, we will use ‘reservoir’ as an overarching term for any of these measurements, including measurements in individuals not on ART, in whom the reservoir consists of both latently infected cells and cells involved in ongoing viral replication. Studies were required to either compare reservoir size across subtypes or report reservoir measurements alongside subtype data at the individual or subtype-group level. No restrictions were applied with respect to study design or geographical area. Any disparities during the study selection process were resolved by consensus.

### 2.3 Data extraction

Two authors (MSW and AANT) independently extracted data from each eligible study using a data extraction form in Microsoft Excel developed in consultation with an information specialist and other co-authors. Data were extracted on: general article information; sample size; study design; geographic location of study; baseline characteristics of participants; duration of follow-up; reservoir quantifications; assays used for reservoir quantification, and viral subtyping. When available, individual-level data were additionally extracted on HIV-1 subtype, reservoir outcomes, and potential confounders. In cases where numerical data were reported only in graphical form, summary statistics were extracted using WebPlotDigitizer (version: 5.2; Ankit Rohatgi) and recorded accordingly. For each included study, data extraction was performed by one author and independently verified by the second author.

Viral subtype nomenclature was harmonized according to the Los Alamos HIV Sequence Database guidelines (42), and subtype labels were adjusted as necessary to ensure consistency across studies. For clarity, analyses in the main text focus on the most abundant HIV-1 subtypes globally. Six subtype categories were defined: A (including A and A1), B, C, D, CRF01_AE, and CRF02_AG. The category “Other” was used to denote all subtypes not explicitly reported in a given study, including subtypes represented by fewer than three individuals and those outside the main categories, such as F, G, H, other circulating recombinant forms (CRFs), unique recombinant forms (URFs), and mixed-subtype co-infections. “Unknown” denotes samples for which subtype information was not analyzed or not reported in the included study.

Disease stages were grouped into acute, early, and chronic infection, using study-defined classifications where available. Otherwise, we defined acute infection as Fiebig I–II, early infection as <6 months post-infection, and chronic infection as >6 months post-infection.

### 2.4 Quality assessment

The quality of included studies was assessed with respect to their suitability for comparing reservoir quantification outcomes across subtypes, using an adapted version of the Joanna Briggs Institute (JBI) critical appraisal tool for cohort studies (43). The assessment focused on factors most relevant to data extraction and interpretation, including identification of potential confounders, as well as the validity and reliability of HIV-1 reservoir quantification and subtyping methods. For the studies that performed statistical comparisons between subtypes, the appropriateness of the statistical analyses was also evaluated. Quality assessment for each article was conducted by one author (MSW or AANT) and independently verified by the second author.

### 2.5 Data synthesis and analysis

Depending on data availability, continuous variables were summarized for each subtype group using the median and interquartile range (IQR), minimum and maximum values, and/or the mean. Reservoir quantification data were log_10_-transformed where necessary to standardize units across studies. Any additional transformations are explicitly indicated. When data from individual participants were available, summary statistics were calculated and verified against values reported in the original publications. Mean values were calculated on log_10_-transformed data, excluding samples reported as undetectable. Measurements reported with an upper detection limit were included using the reported bound (e.g., values reported as <50 were treated as 50). Ordinal data were summarized using percentages, while all other data were summarized descriptively. For longitudinal studies, we present summary statistics separately for each time point. Studies were grouped and analyzed according to the assessed reservoir compartment (**Table 1**) and participant ART status. To indicate the direction of effect, within-study statistical comparisons of reservoir size between subtypes were summarized. Analyses and visualizations were performed using R Statistical Software (44).

## 3 Results

### 3.1 Study selection

**Figure 1:**
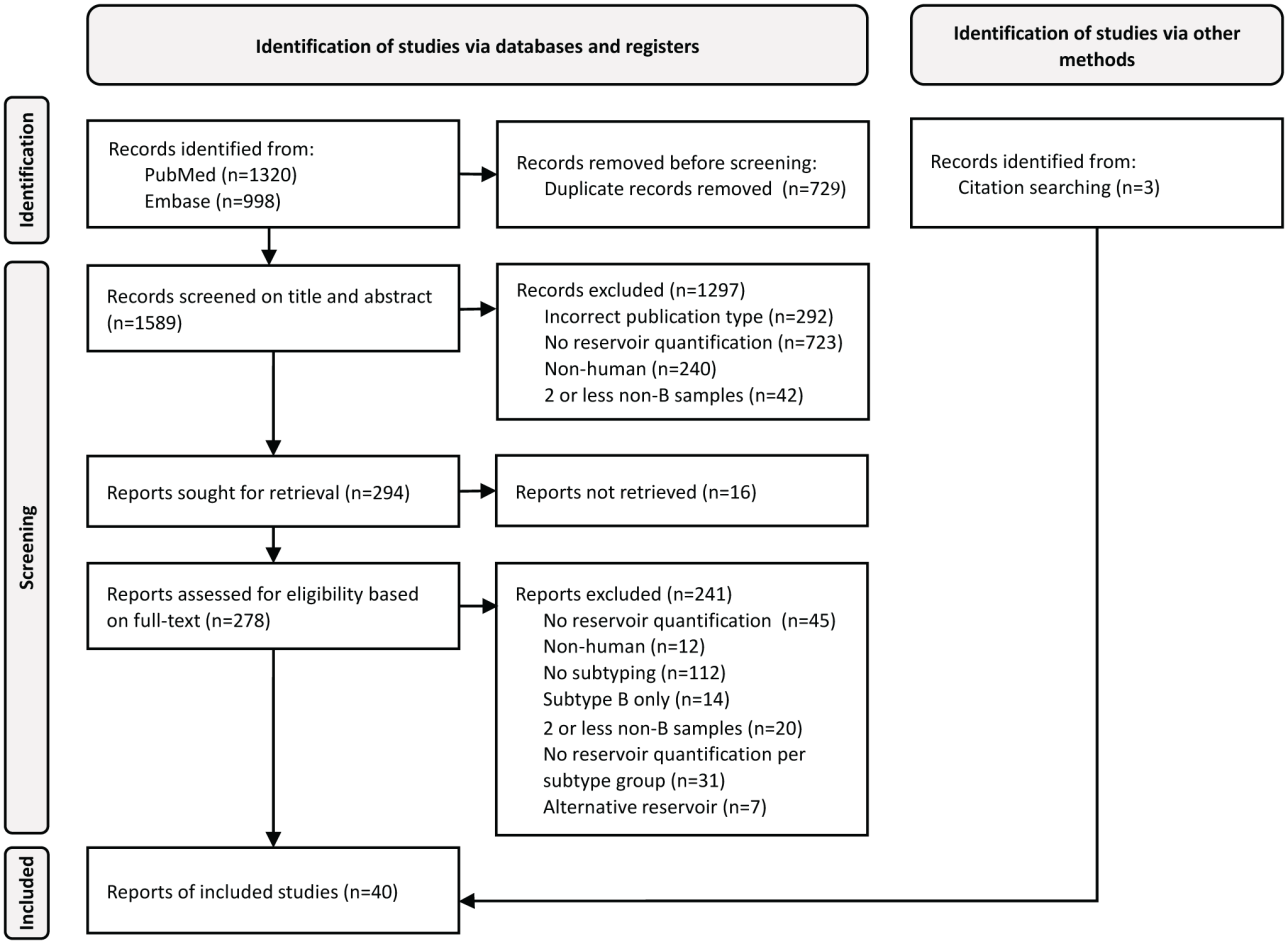
PRISMA flow diagram of the study selection. Exclusion reasons are listed in the order of application.

This systematic literature search identified 2318 records, of which 1589 were unique (**Figure 1**). After full text screening, 40 articles were included in the final analysis (**Table 2**; **Supplementary Table 2** for detailed extracted data), of which three articles were identified through reference searching (6, 45, 46). Among excluded articles, subtypes were not reported in about half of the articles that quantified the HIV-1 reservoir. Among the articles reporting both subtype and reservoir quantification, 31 did not compare reservoir quantification across subtypes or did not report quantifications stratified by individual or by subtype group.

**Table 2:** Participant characteristics and HIV-1 subtypes of the 40 included studies, sorted by publication date.

Study	Number of participants	Location of sampling	Age*	Sex^†^	ART status and duration of viral suppression (years)	Measured	Time points^‡^ quantifications	Subtype and corresponding number of participants
Casabianca 2007 (47)	73	Italy	–	–	- (on ART)	Total	1	B: 65, C: 2, F: 2, CRF01_AE: 2, CRF02_AG: 2
Kondo 2009 (48)	18	Japan	–	–	Median: 0.3, IQR: 0-2.6	Total	1	B: 8, CRF01_AE: 8, F: 2
Troude 2009 (49)	903	France	Mean: 35	M: 756 F: 138	Not on ART	Total	1	B: 662, CRF02_AG: 115, Other: 126
Demetriou 2010 (50)	161	Europe**, Israel	Median: 34, IQR: 29-42	M:116 F: 28, Unknown:17	Not on ART	Total	1	A: 23, B: 103, C: 14, D: 1, F1: 1, G: 7, CRF02_AG: 6,CRF01_AE: 4, C/H/K: 1, CRF02_B: 1
Heeregrave 2010 (51)	5	Netherlands	–	–	- (on ART)	Total	1	C: 2, D: 1, F:1, A:1
Antoniadou 2014 (52)	98	Greece	Median: 34, IQR: 27-41	M: 88, F: 10	Not on ART	Total	1	A:42, B:44, C: 3, F1: 1, G:1, CRF02_AG: 4, CRF04_cpx: 2, CRF01_AE: 1
Zhang 2014 (53)	15	China	–	M: 15, F:0	Not on ART	Total, Integrated	4	CRF01_AE: 15
Du 2016 (54)	19	China	Median: 32, IQR: 30-35	M: 19, F:0	Median: 0.5, IQR: 0.5-0.5	Total	1	B:12, C:7
Jiao 2017 (55)	7	China	Median: 32, IQR: 28-37	M: 7, F: 0	–	Total, Integrated	1	CRF01_AE: 4, CRF07_BC: 3
Prodger 2017 (45)	121	Uganda, United States	Median: 43, IQR: 32-54	M: 23 + 38 F: 47 + 13	- (on ART)	Replication competent	1	D: 40, A:11, Recombinant (A/D):7, B:15, unknown: 48
Wang 2017 (56)	17	China	Median: 34, IQR: 28-41	M:16, F:1	Not on ART at baseline	Total	4	CRF01_AE: 9, Recombinant (CRF07_BC/08_BC): 8
Kuhn 2018 (57)	146	South Africa	Median: 5, IQR: 4-5	M: 64, F: 82	–	Total	1	C: 146
Lu 2018 (58)	48	China	–	M:48, F:0	Not on ART at baseline	Total	2	CRF01_AE:22, B: 10, CRF07_BC: 16
Rutsaert 2018 (59)	91	Belgium	–	–	–	Total	1	B:10, CRF01_AE: 10, CRF02_AG: 10, A:10, C:10, F1:11, G: 8, Other:5, D: 5, CRF06_cpx: 5, H: 3, F2: 3, J: 1
Bachmann 2019 (6)	1057	Switzerland	Median: 40, IQR: 35-50	M: 803, F: 254	Median: 1.1, IQR: 0.9-1.4	Total	3-4	B:627, CRF01_AE: 65, CRF02_AG: 41, A: 39, C: 36, Recombinant:27, D:12, F: 12, G: 12, CRF06_cpx: 2, CRF11_cpx:2, CRF18_cpx:1, CRF12_BF: 1, CRF19_cpx:1, CRF20_BG: 1, H: 1, unknown: 177
Crowell 2019 (60)	18	Thailand	Median: 28, IQR: 25-41	M: 18, F:0	Not on ART at baseline	Total, Integrated, Inducible	2	CRF01_AE: 12, B:4, co-infection (CRF01_AE and B):1, recombinant (CRF01_AE/B/C):1
Lee 2019 (25)	4	South Africa	–	F: 4, M:0	Not on ART at baseline	Total, Intact	1-4	C:4
Leite 2019 (61)	10	Brazil	Median: 28	M: 10, F: 0	1	Total	2	F1: 2, C:2, B:6
Omondi 2019 (62)	30	Canada	–	M: 30, F:0	0	Total, Replication competent	2	B:25, CRF01_AE:2, G:3
Bertoldi 2020 (63)	12	Netherlands	–	–	Median: 2, IQR: 2-2.4	Total, Inducible	1	B: 3, C: 9
Gunst 2020 (46)	59	Denmark, United Kingdom	Median: 36, IQR: 28-47	F: 5, M: -	Not on ART	Intact	1	A: 2, B: 28, C: 5, D: 3, F: 1, G: 1, CRF01_AE: 14, CRF02_AG: 3, CRF47:1, Other: 1,
Katusiime 2020 (64)	8	South Africa	Median: 5, IQR: 2-9	F: 6, M: 2	Median: 5.2, IQR: 4.9-6.6	Total, Intact	1	C:8
Lyu 2020 (65)	373	China	Median: 35, IQR: 27-43	M:263, F:110	Not on ART at baseline	Total	4	CRF01_AE: 188, Other: 185
Ruhanya 2020 (66)	147	South Africa	Mean: 32	M: 24, F: 121	Some on ART	Total	1	C: 147
Koofhethile 2021 (67)	15	Botswana	Median: 16, IQR: 16-16	M: 10, F: 5	Median: 15, IQR: 14.5-15	Total, Replication competent	1	C: 15
Mehta 2021 (68)	12	India	Median: 37, IQR: 32-41	M: 7, F: 5	Median: 0.5, IQR: 0.4-0.5	Total, Inducible	1	C: 12
Joussef-Piña 2022 (69)	112	Uganda, United States	–	M: 23 + 42, F: 20 + 7	- (on ART)	Total, Inducible	1	A:19, C:3, D:40, B:50
Hossain 2023 (70)	34	Netherlands	–	F: 25, M: 7	- (on ART)	Total, inducible	1	C:19, B:15
Huang 2023 (71)	10	United States	Median: 16, IQR: 14-17	F: 8, M: -	Median: 8.2, IQR: 5-12.7	Total	1	B:7, C:1, Recombinant (A/G):1, Recombinant (A/E):1
Koofhethile 2023 (72)	9	Mozambique	Median: 0, IQR: 0-0	–	–	Total, intact	3	C:9
Trémeaux 2023 (73)	35	France	Median: 35, IQR: 28-42	–	Median: 3.2, IQR: 0-6.4	Total, Intact	1	B: 26, Recombinant (B/F):1, C: 1, CRF01_AE: 2, CRF11_cpx: 1, CRF13_cpx: 2, H: 1, Recombinant(A/)?: 1
Fuchs 2024 (74)	312	France	Median: 47, IQR: 39-56	F: 122, M: -	Median: 10, IQR: 5.6-17	Total, Integrated	1	A:33, B:66, C:25, D:12, F:9, G: 22, CRF01_AE: 23, CRF02_AG: 31, CRF06_cpx: 12, CRF15_01B: 5, CRF11_cpx:15, CRF13_cpx:5, CRF18_cpx: 8, CRF22_01A1: 8, CRF30_0206: 5, CRF36_cpx: 5, other: 28
Lee 2024 (75)	23	Uganda	Median: 42, IQR: 38-45	M: 7, F: 16	Median: 9, IQR: 6-11	Total, Intact, Replication competent	1	A1:4, C:1, D:9, Recombinant (A1/D):9, Recombinant (A1/D/C):1
Buchholtz 2024a (76)	5	Netherlands	–	–	- (on ART)	Intact	1	C: 5
Buchholtz 2024b (77)	82	South Africa	–	F: 51, M: 31	Not on ART at baseline	Total, Intact	3	C:82
Ambrosioni 2025 (78)	64	Spain	Median: 32	M: 60, F: 4	Not on ART at baseline	Total, Intact	2	B: 39, A: 5, CRF02_AG: 8, Recombinant (B & F): 4, Uknown:1, Other:7
Ehrenberg 2025 (79)	52	Thailand, United States	–	M: 52, F:0	0.9	Total, Integrated	1-2	CRF01_AE:12, Recombinant (CRF01_AE/B): 2, B:17, unreported: 21
Mchantaf 2025 (80)	7	France	Median: 27, IQR: 23-28	M: 5, F: 2	Median: 17, IQR: 15.5-23	Total	1	CRF02_AG:2, C:1, B:4
Reddy 2025 (81)	35	South Africa	Mean: 21, Range: 18-24	F: 35, M: 0	Median: -1.2, IQR: -2.1--1	Total, Intact	3-5	C: 35
Tschumi 2025 (31)	62	Switzerland	–	F: 13, M: 49	–	Intact	1	CRF01_AE:8, CRF02_AG: 2, A:6, B:34, C:4, F:4, G:3, Others:1

*Age and duration of viral suppression are reported at the first recorded time point. A dash (“-”) indicates unreported data.

† Abbreviations for the sex column: “F” = Female, “M” = Male.

‡ In the time point column, a range (e.g., “3-5”) indicates that the number of time points differed across patients. A “1” denotes a cross-sectional measurement; other studies had longitudinal measurements of reservoir outcomes.

** Europe: Belgium, Croatia, Cyprus, Greece, Israel, Luxembourg, Slovenia, Spain.

### 3.2 Risk of bias

**Figure 2:**
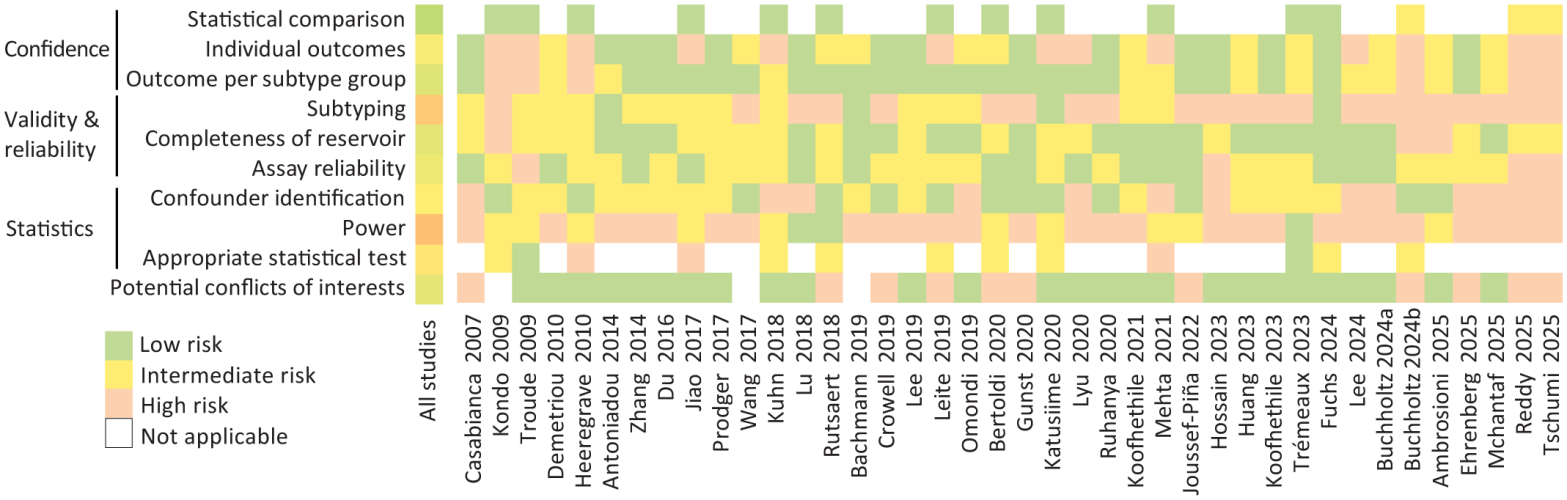
Risk of bias assessment across included studies. Green indicates low risk, yellow indicates intermediate risk, and pink indicates high risk. Items rated as not applicable (n.a.) are shown in white and were excluded from scoring. Assessment criteria and study-level assessments are provided in **Supplementary Table 2** (data sheet).

The quality of articles was assessed based on their suitability for comparing HIV-1 reservoir outcomes and dynamics across subtypes. Across all domains assessed, the risk of bias was high in the majority of studies (**Figure 2**). The main sources of bias were low statistical power, driven by small sample sizes per subtype, and limited validity and reliability of subtyping methods. Specifically, only 9 studies included at least 50 samples in two or more subtype groups, and 22 studies did not clearly report the subtyping methods. Among studies performing statistical comparisons across subtypes, risk of bias arose from a lack of adjustment for confounders and poor reporting of statistical methods. Overall, reporting of confounders was often insufficient, and individual-level data were only available in 24 out of 40 studies (**Supplementary Table 2** for individual-level data). These findings underscore the need for improved study design and reporting to enable robust comparisons of HIV-1 reservoir outcomes across subtypes (see section 4.2, recommendations).

### 3.3 Demographical characteristics

**Figure 3:**
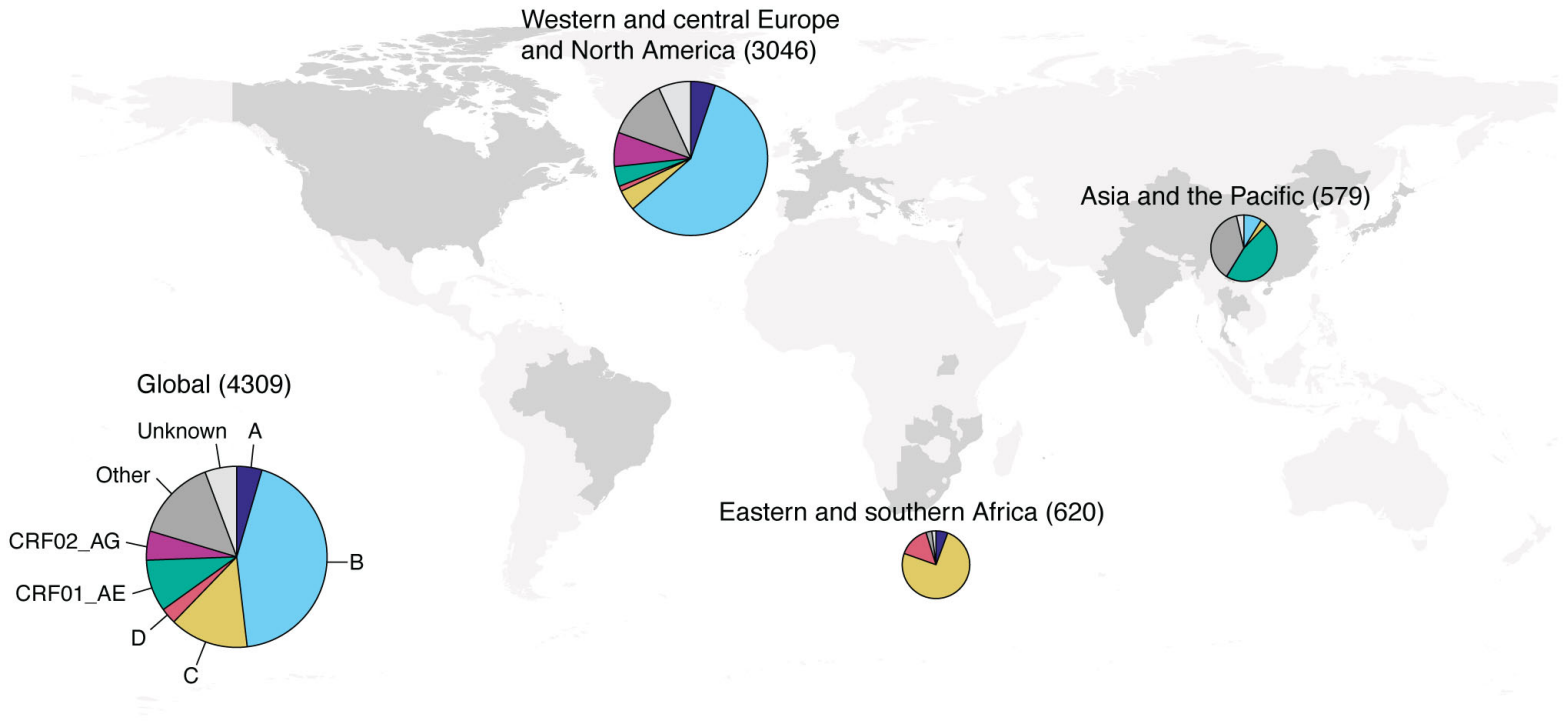
Participant sampling locations and HIV-1 subtype distribution by UNAIDS region. Darker gray shading on the map indicates countries from which participants in the included studies were sampled. Pie charts display HIV-1 subtype proportions for UNAIDS regions with more than 30 participants. Regions with fewer than 30 participants are not shown in the pie charts (Latin America: 10; Eastern Europe and Central Asia: 26; Middle East and North Africa: 18). The six most prevalent subtypes are shown. “Other” denotes all remaining subtypes and those labeled as “other than subtype X” by the study. “Unknown” denotes samples for which subtype information was not analyzed or not reported.

**Figure 4:**
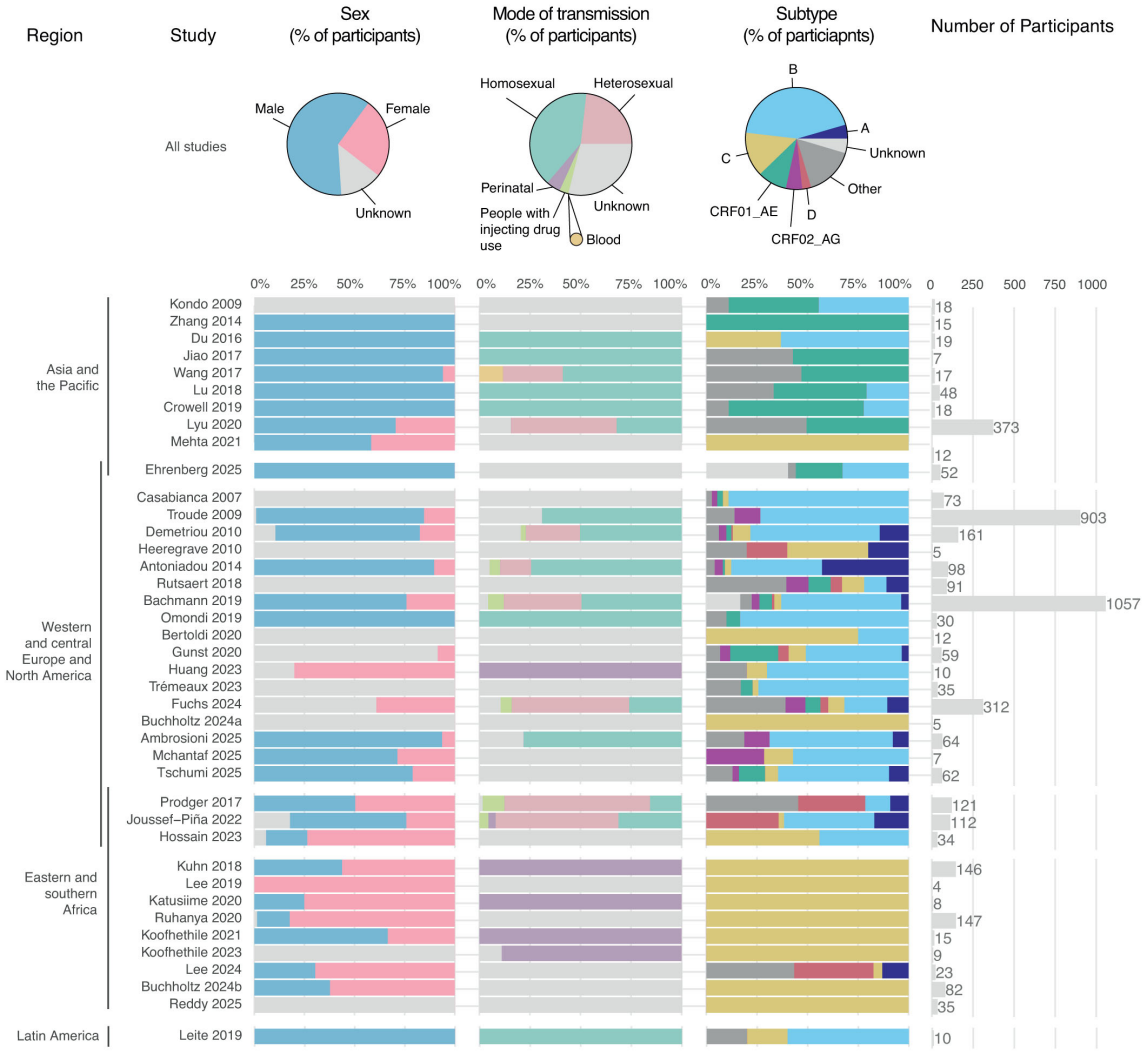
Demographics of included participants. Bar charts display study-level percentages for sex, modes of transmission, subtypes, and the total number of participants. Pie charts summarize these characteristics across all 4309 included participants. Studies are grouped by UNAIDS region and sorted by year.

Available data from a total of 4309 participants were included in this systematic review (**Table 2**), not accounting for some overlaps across six articles (6, 25, 31, 45, 75, 76, 81). The number of participants varied across studies (median: 35, IQR: 12 - 93). Most studies and participants were from North America or Europe, followed by Eastern and Southern Africa and Asia (**Figure 3**). The majority of the participants were male (>61%). Female participants were more frequently included in studies from Southern Africa (**Figure 4**). Along the same lines, the mode of transmission reflected the sampling location. Among participants from North America, Europe, and Asia, homosexual contact predominated, whereas in Eastern and Southern Africa, heterosexual contact and perinatal transmission were more common (**Figure 4**). Most studies focused on adults, while three studies included young children (57, 64, 72), and two studies included perinatally transmitted adolescents (67, 71). Reservoir studies in pediatric acquired HIV-1 are scarce, although infection early in life, often before immune system maturation, may influence reservoir establishment and persistence (82).

### 3.4 Clinical characteristics

Most studies included peripheral blood samples of individuals on ART, although the duration of viral suppression varied. Most reservoir measurements were conducted within 1.5 years of suppression (22 time points), including 18 within the first year of which 7 were within the first six months; the maximum reported duration of suppression was 24 years (**Supplementary Figure 1**). In studies reporting ART initiation stage, initiation occurred during acute, early, and chronic infection in 8, 3, and 5 studies, respectively, while 4 studies included individuals with mixed ART initiation stages (**Supplementary Figure 1**). Among individuals not on ART, duration of infection and disease stage varied and were frequently unreported (**Supplementary Table 2**).

### 3.5 Subtypes

Although study identification and selection were designed to include studies on non-B subtypes, subtype B remained the most frequently reported, as it represented the majority of participants in many included studies. Subtype B predominated in studies conducted in North America and Europe, while subtype C was most common in Eastern and Southern Africa, and subtype CRF01_AE in Asia (**Figure 3**). For 5.7% of the participants, the subtype was unknown. Among the studies reporting subtyping methods, two reported (near) full-length sequencing (6, 60), while 19 relied on PCR-based sequencing of specific regions of the viral genome, most commonly targeting the HIV-1 *env* gene (**Supplementary Figure 2**; **Supplementary Table 2**). In 6 of these 17 studies, subtype assignments were further confirmed by sequencing an additional genomic region (45, 52, 53, 65, 69, 75).

### 3.6 Total HIV-1 reservoir

**Figure 5:**
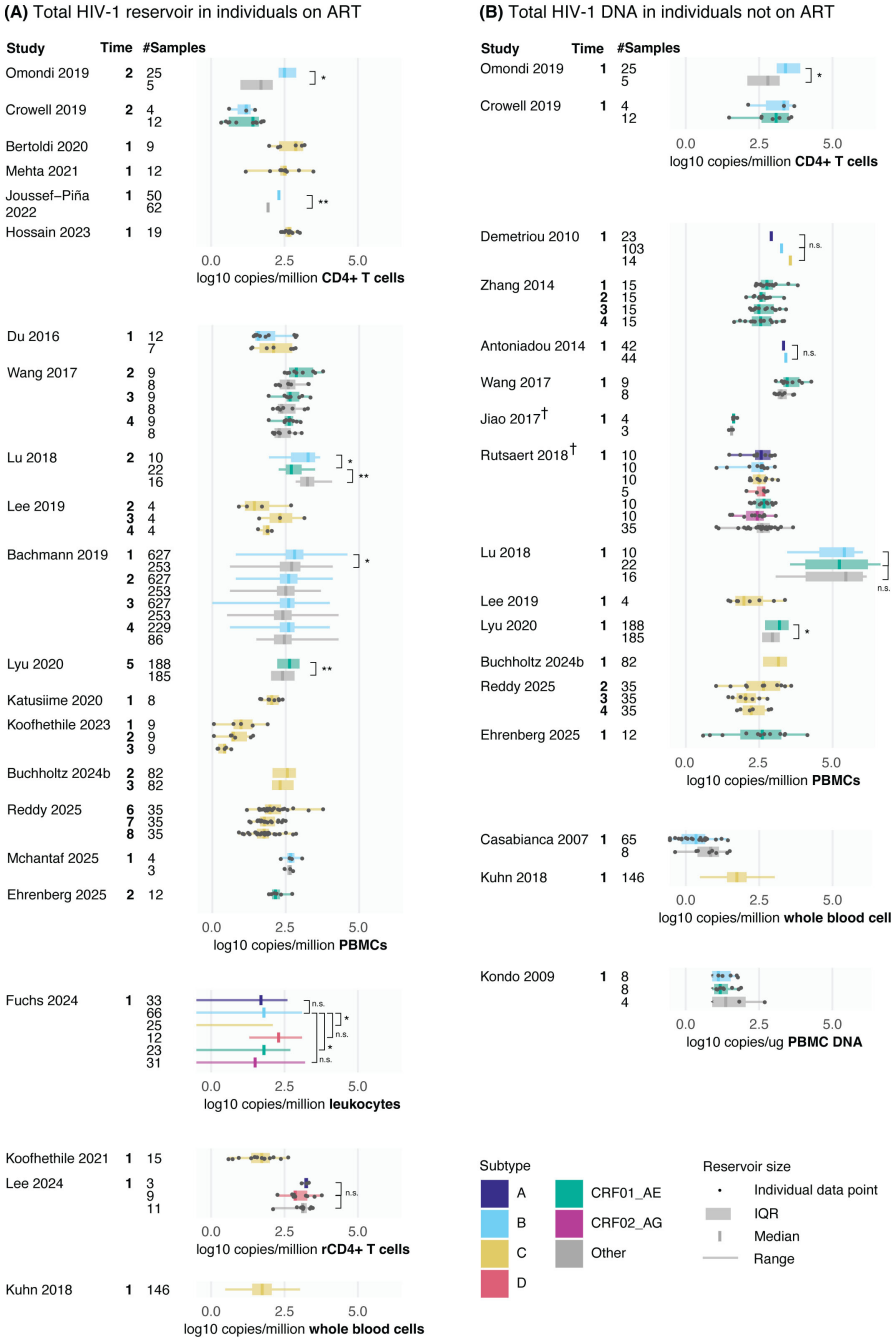
Total HIV-1 DNA quantifications by study and subtype. **(A)** Individuals receiving ART **(B)** Individuals not on ART or with an unknown ART status, stratified by study, time point, and subtype. Studies with unknown ART status are denoted with ‘†’. Boxplots (colored by HIV-1 subtype) show reservoir measurements (interquartile range (IQR), median, mean, and/or minimum-maximum range, depending on the availability of summary statistics), for subtype groups with 3 or more samples per study, with individual data points overlaid when available. Results are separated into subplots by reported units. Brackets (“]”) indicate statistical comparisons by the authors of the included study (*p < 0.05; **p<0.005; “n.s”, non-significant). “Other” denotes all remaining subtypes and those not explicitly reported in a given study. Data for unknown subtypes is not shown. For studies that performed several different total HIV-1 DNA assays on the same sample, one assay was shown (Casabianca 2017: pbs-rtPCR, converted from copies per ug cell DNA to copies per million cells; Rutsaert 2019: dd-PCR assay “Schvachsa_2007”; Fuchs 2024: LTR-gag). Trémeaux 2023 is excluded (all non-B samples being post-treatment or elite controllers). For studies that did not report subtype-specific data, results could not be shown in the figure. ‘Time’ refers to the sampling time point in longitudinal studies (**Table 2**).

**Figure 6:**
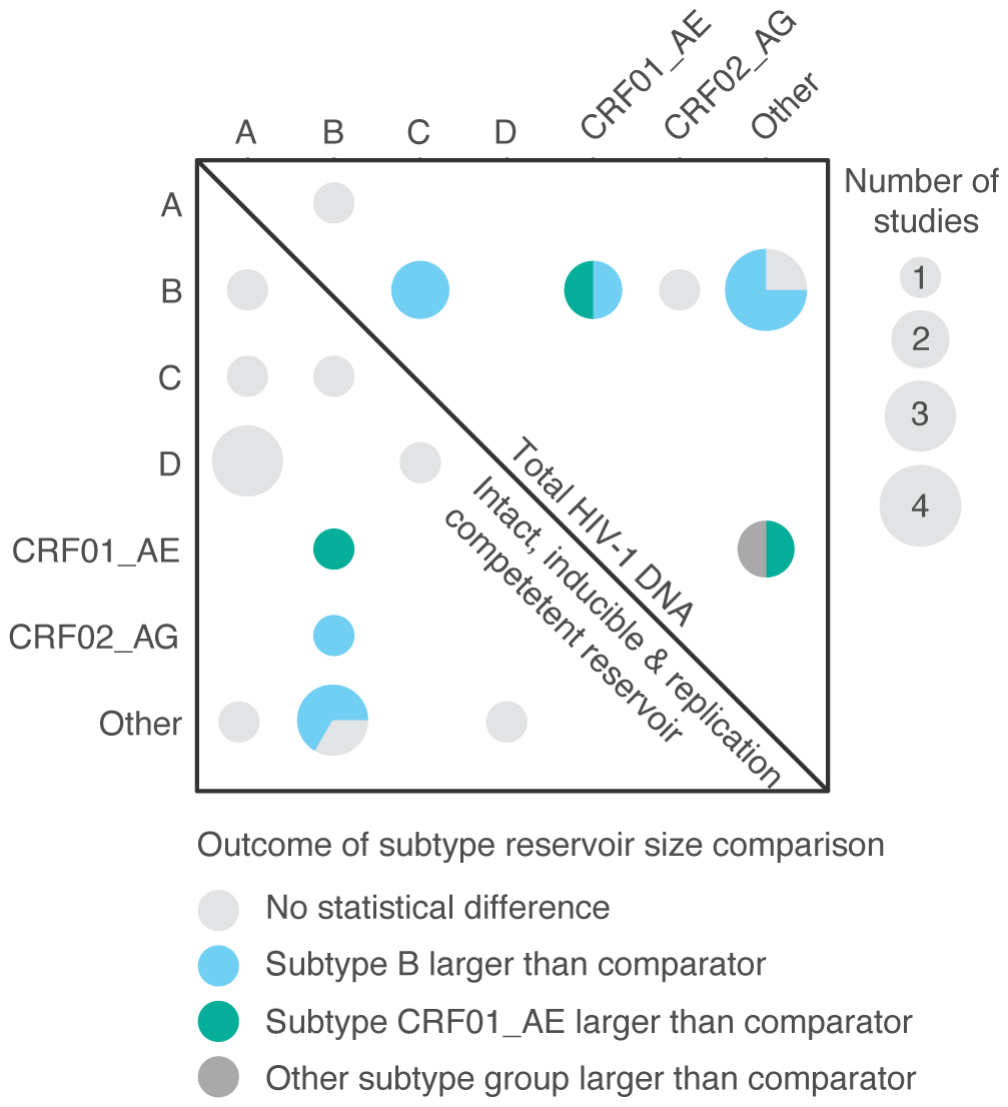
Statistical subtype comparisons. The number and outcome of reservoir size comparisons in individuals on ART with different subtypes. Total HIV-1 is shown in the upper triangle, and the other reservoir measures in the lower triangle.

Total HIV-1 DNA, typically defined as the sum of all intracellular HIV-1 DNA, measured in peripheral blood, was the most commonly reported proxy for the viral reservoir (**Table 1**; **Table 2**; **Figure 5**). We next summarized studies comparing total HIV-1 DNA levels across individuals with different subtypes (the upper triangle in **Figure 6** visualizes the outcomes of total HIV-1 DNA comparisons in individuals on ART), first focusing on comparisons involving subtype B, followed by subtypes CRF01_AE and C.

Among individuals on ART, three studies suggested a larger total HIV-1 DNA reservoir in people with subtype B compared to grouped non-B subtypes (6, 62, 69). Bachmann et al. reported higher reservoir levels in individuals with subtype B at 1.5 years after ART initiation, although this difference was no longer significant after multivariable adjustment (6). Omondi et al. similarly observed a larger reservoir in subtype B at 48 weeks post-treatment (62). Joussef-Piña et al. reported higher reservoir levels among individuals with subtype B when comparing distinct cohorts from Uganda and the United States (62). Underscoring this, Fuchs et al. applied multiple total HIV-1 DNA assays to the same samples and reported that subtype B tended to have a larger reservoir than subtype C, but a smaller reservoir than subtypes A and CRF01_AE, although statistical significance was not consistently observed across assays (74). Differences in detection rates across assays may reflect variation in assay target regions.

In individuals not receiving ART, studies comparing total HIV-1 DNA between subtype B and non-B subtypes yielded mixed results. Omondi et al. reported less total HIV-1 DNA in non-B subtypes (62), whereas Troude et al. reported a larger reservoir in non-B subtypes (49), and two other studies found no difference (50, 52). These inconsistent findings likely reflect substantial heterogeneity across studies, including differences in study design, populations, the specific non-B subtypes examined, and statistical approaches, with some studies not accounting for potential confounders (50, 52).

For subtype CRF01_AE, comparisons across three studies indicated conflicting results (58, 65, 74). In individuals on ART, Lyu et al. reported a larger total reservoir for subtype CRF01_AE compared to non-CRF01_AE (65), as well as Fuchs et al., who found that subtype CRF01_AE had a significantly larger total reservoir than subtype B in one of two assays (74). In contrast, Lu et al. found a smaller reservoir in individuals with subtype CRF01_AE, when compared to subtype B or CRF07_BC (58). In individuals not on ART, Lyu et al. reported a higher reservoir in subtype CRF01_AE (65), whereas Lu et al. found no difference (58). These discrepancies may reflect differences in the comparator subtypes and statistical approaches, but unclear reporting of these factors in some studies limits interpretation.

Two studies reported a smaller reservoir in ART-treated individuals with subtype C compared with subtype B (6, 74), whereas two others could not confirm a significant difference between C and other subtypes in treated individuals (69) and in untreated individuals (50). Although subtype C was included in 21 additional studies, most were limited to either small numbers of participants with subtype C or subtype C–only cohorts, precluding comparisons with other subtypes. Even among these studies, results vary, possibly due to differences in age, timing of treatment initiation, and assay variability across the cohorts.

### 3.7 Integrated reservoir

Five studies assessed both the integrated and total HIV-1 reservoir (53, 55, 60, 74, 79), but only the study by Fuchs et al. included subtype comparisons (the lower triangle in **Figure 6** visualizes subtype comparisons of the integrated, intact, inducible, and replication-competent reservoir in individuals on ART). This study found a smaller integrated reservoir for subtype C and CRF02_AG, when compared to subtype B. In contrast, subtype CRF01_AE showed a larger integrated reservoir than subtype B. For subtype C and CRF01_AE, these differences mirrored those observed for the total HIV-1 DNA reservoir (**Supplementary Figure 3**).

### 3.8 Intact reservoir

Eleven studies assessed the intact reservoir, mostly using near full-length sequencing or PCR-based assays like IPDA (**Supplementary Figure 3**, **Supplementary Table 2**). Six of these studies included only participants with subtype C (25, 64, 72, 76, 77, 81), and found varying levels of intact provirus, ranging from 0% in acutely treated individuals 12 months post-treatment (81), 1% in early-treated children between 19–36 months old (64), 7% in acutely treated perinatally transmitted infants (72), and 14% in chronically treated individuals 12 months post-treatment (81).

Three studies compared intact reservoir measurements across multiple subtypes (31, 46, 75) (**Figure 6**). Tschumi et al. found a lower detectability of intact provirus in subtypes CRF01_AE, C, and F compared to subtype B (31). No such difference was observed when comparing subtypes CRF02_AG, A, and G to subtype B. While interesting, this study included both treated and untreated individuals, which complicates the interpretation of these outcomes. Two other studies found no significant differences in the proportion of intact proviruses (46, 75). Lee et al. observed comparable levels between subtypes A1, D, and A1/D recombinants (75), and Gunst et al. reported no differences between subtype B and non-B subtypes overall (46).

### 3.9 Inducible and replication competent reservoir

Five studies used RNA-based assays to measure the inducible reservoir (60, 63, 68–70), and four studies assessed the replication-competent reservoir (**Table 1**; **Supplementary Figure 3**) (45, 62, 67, 75). Three of these studies included multiple subtypes and performed statistical comparisons (45, 62, 69) (**Figure 6**). Omondi et al. reported a smaller replication-competent reservoir in individuals with non-B subtypes compared to subtype B (62). Similarly, Joussef-Piña et al. observed a smaller inducible reservoir in individuals with non-B subtypes from a Ugandan cohort compared to individuals with subtype B from a United States cohort (69). Notably, when assessed within the Ugandan cohort, no association between subtype and inducible reservoir size was observed across subtypes A, C, and D. Similarly, Prodger et al. reported a threefold smaller replication-competent reservoir in chronically treated Ugandan individuals with non-B subtypes compared to United States individuals with subtype B (45).

### 3.10 Correlations between reservoir measures

Nineteen studies assessed multiple measures of the HIV-1 reservoir, of which four evaluated the correlation between these measurements in a subtype-specific context (31, 63, 68, 70). For subtype C, Bertoldi et al. and Mehta et al. reported a positive correlation between the total and the inducible reservoir (63, 68). In contrast, Hossain et al. did not find a significant correlation between these two measures in subtypes B nor C (70). Only one study compared the correlations across subtypes: Tschumi et al. reported that the correlation between intact and total HIV-1 DNA was similar when considering all subtypes combined or subtype B alone (31).

### 3.11 Reservoir dynamics

**Figure 7:**
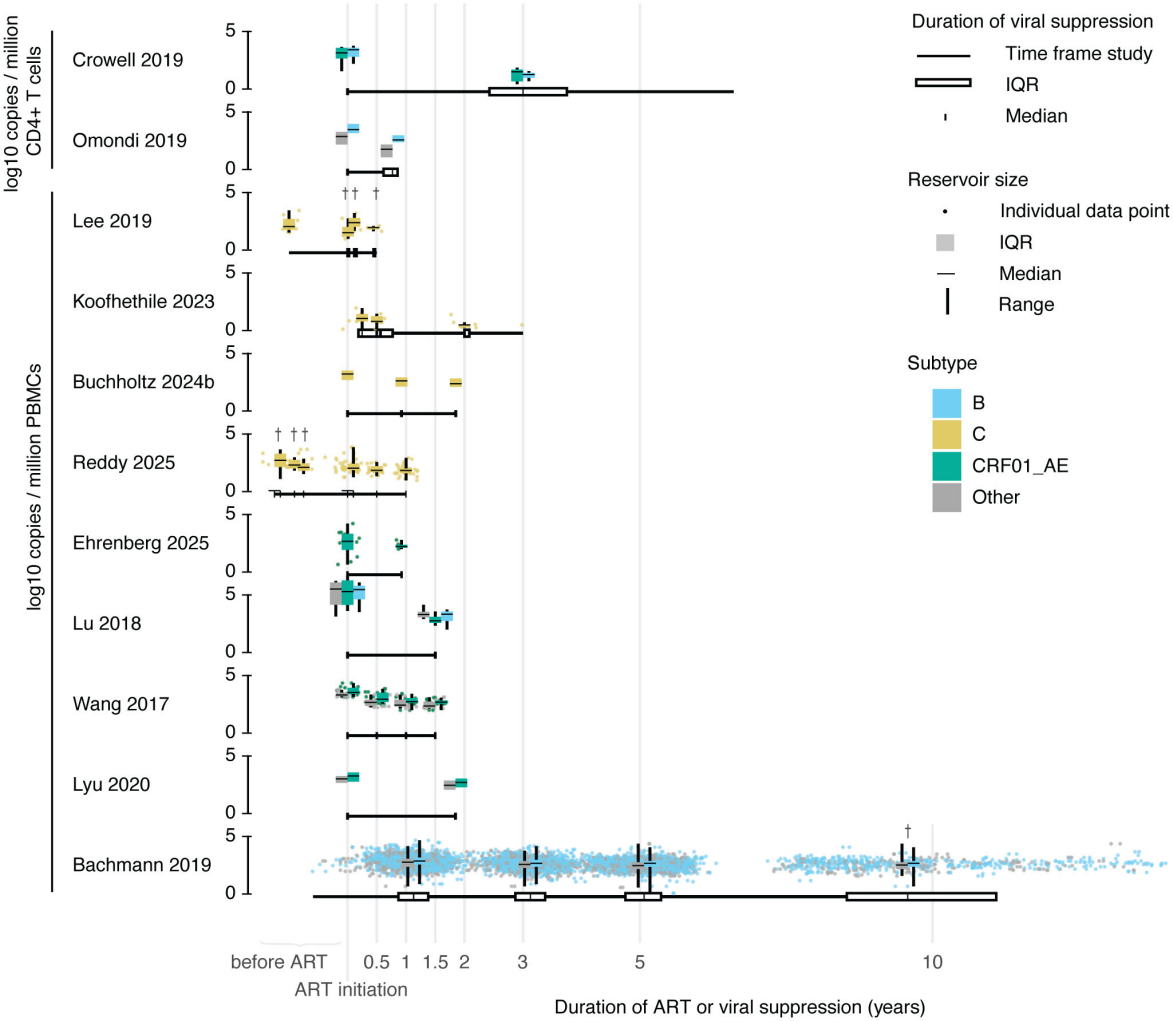
Longitudinal total HIV-1 DNA measurements in individuals receiving ART. The timeline illustrates the frequency of reservoir quantifications and intervals relative to the initiation of ART or onset of viral suppression. The solid line represents total study duration. “†” indicates that only a subset of participants was included at this specific time point.

Fourteen studies measured the HIV-1 reservoir longitudinally, with most participants sampled at multiple time points. The intervals between sampling ranged from 40 days to 10 years. Of these, four studies examined subtype-specific differences in the decay of total HIV-1 DNA (6, 58, 62, 65). Omondi et al. and Bachmann et al. reported slower decay in people with subtype B compared to non-B during the first 40 weeks on ART and between 1.5 and 4.5 years on ART, respectively (6, 62). Both Lu et al. and Lyu et al. reported slower decay for subtype CRF01_AE compared to non-AE, although this difference reached statistical significance only in Lu et al. (58, 65). In addition, four studies reported longitudinal data on reservoir dynamics for individuals with subtype C (25, 72, 77, 81). As these studies included only subtype C, comparisons with other subtypes could not be made. However, all showed a decrease in reservoir size over time (**Figure 7**).

## 4 Discussion

### 4.1 Main findings

In this systematic review, we compiled an overview of the studies quantifying the HIV-1 reservoir in peripheral blood among individuals with non-B subtypes and created a repository of reservoir outcomes with associated metadata. Overall, we identified 40 studies reporting reservoir quantification in people with non-B subtypes, providing initial insights into subtype diversity in HIV-1 reservoir research (**Table 2**). In terms of participant characteristics, we found that viral subtype, biological sex, and mode of transmission largely reflected the geographical location of the study cohorts (1) (**Figure 4**). The diversity in geographical and cohort characteristics between included studies supports caution in cross-cohort comparisons.

Across individual studies, comparisons between treated individuals with subtype B and pooled non-B subtypes generally indicated a trend toward a larger reservoir in individuals with subtype B (**Figure 6**). This pattern was most evident for total HIV-1 DNA (6, 62), particularly in comparison with subtype C (6, 74). Similarly, the intact reservoir was found to be larger in individuals with subtype B, compared to subtypes C, CRF01_AE, and F (31). Also, the inducible and replication-competent reservoir was reported to be larger in treated individuals with subtype B (45, 62, 69). However, these results need to be interpreted with caution, as many comparisons were based on geographically and demographically distinct cohorts, limiting direct comparability (45, 69).

While subtype B was most frequently included in statistical comparisons of reservoir size, several studies also investigated individuals with subtype CRF01_AE, both treated and untreated. However, these analyses yielded inconsistent findings (31, 58, 65, 74).

### 4.2 Knowledge gaps in existing literature and recommendations for future research

Overall, the available data suggest a tendency toward a larger HIV-1 reservoir in individuals with subtype B. Evidence of this, however, is limited, and additional studies are needed. Important knowledge gaps persist across several key domains. First, studies are constrained by small and imbalanced study populations, with underrepresentation of non-B subtypes. Second, methodologically, most analyses rely on total HIV-1 DNA in peripheral blood, with limited assessment of integrated, intact, inducible, or replication-competent reservoirs. Third, longitudinal data on reservoir dynamics across non-B subtypes are scarce, and statistical comparisons are often underpowered or insufficiently adjusted for confounding factors. Fourth, inconsistent reporting of subtype, individual-level data, and key metadata limits cross-study comparability. Together, these gaps form the basis for the recommendations outlined below (**Box 1**), which aim to promote more standardized, comprehensive, and cross-study comparable approaches to HIV-1 reservoir research.

Box 1 **Recommendations for future studies comparing the HIV-1 reservoir across subtypes.**

**Table:** 

Study design and population	Sample size and subtypes	Perform power analyses to ensure adequate sample size for subtype comparisons.	Recommended
	Comparability	Ensure comparability of key participant characteristics across subtypes.	Recommended
	Multiple subtypes	Include participants representing different HIV-1 subtypes.	Recommended
Laboratory and analytical methods	Subtype characterization	Use standardized methods that evaluate multiple genomic regions to ensure accurate subtype identification.	Urgent
	Reservoir assay standardization	Use standardized multi-subtype reservoir assays, or cross-validate assays using subtype-specific reference panels.	Strongly recommended
Statistical analysis	Statistical comparison	Perform statistical comparisons of outcomes across subtypes adjusting for relevant confounders.	Recommended
Reporting and data availability	Subtypes	Classify subtype according to Los Alamos guidelines and report results per subtype.	Urgent
	Individual data	Report individual-level data where possible; otherwise provide summary statistics with appropriate measures of uncertainty.	Urgent
	Metadata	Report key metadata at time of sampling: current ART regimen, ART duration/viral suppression duration, and plasma viral load or suppression status.	Urgent
		Report key participant characteristics: ART status, ART duration, disease stage (at ART initiation) or CD4 nadir	Urgent
		Report key participant characteristics: sex, age, ethnicity/geographic origin, co-infections, and therapy history	Recommended

#### 4.2.1 Study design and population

Our risk-of-bias analysis identified limited statistical power for subtype comparisons in many studies. We recommend that studies include sufficiently large and comparable groups across subtypes to enable valid comparisons and adjustment for confounders, although we recognize that the complexity and costs of assays may be limiting factors. Among these groups, key confounding factors must be sufficiently balanced across subtypes to allow meaningful statistical control. Comparisons between cohorts from the United States and Uganda (45, 69) illustrate the challenge of disentangling subtype-specific effects from correlated demographic, clinical, and geographic factors. Where feasible, head-to-head subtype comparisons are preferred over pooled non-B analyses. Sample size requirements should be determined through prospective power calculations in consultation with a biostatistician. Moreover, while it is self-evident that inclusion of participants representing different HIV-1 subtypes is required to compare reservoir sizes across subtypes, this remains a critical consideration, as cross-cohort comparisons have proven challenging. Although recruiting diverse subtypes can be particularly difficult in regions where a single subtype dominates, their inclusion improves the generalizability of the study outcomes. Collaboration can facilitate shared and efficient use of samples.

#### 4.2.2 Laboratory and analytical methods

We urgently recommend the systematic characterization of HIV-1 subtypes, even when a single subtype is expected based on geographic context. Retrospective subtyping may also be feasible using stored samples or existing sequence data. Studies should clearly report the methods and genomic regions used for subtype determination, whereas in more than half of the studies in our dataset (22/40) this was unclear.

Near full-length genome sequencing would reduce misclassification and improve detection of recombinant forms, as demonstrated by Lee et al. (75). They identified additional inter-subtype recombinants missed by previous approaches. However, cost constraints may limit its use in resource-limited settings. As a pragmatic alternative, we recommend sequencing at least two genomic regions to enable detection of potential recombinant forms.

In our dataset, a wide range of assays targeting different genomic regions were used to quantify total HIV-1 DNA, likely contributing to variability across studies. Differences among the >60 available assays (59) limit cross-study comparability. In addition, assays not optimized for non-B subtypes may underestimate reservoir size, introducing bias (70, 76). We therefore strongly recommend the use of validated pan-subtype assays for total HIV-1 DNA quantification, with available assays for non-B subtypes summarized by Sinay & Tiggeler et al. (83). More broadly, assay standardization across the field is needed, considering reliability, cross-subtype performance, scalability, and accessibility. The development and use of reference panels for cross-validation, like those used in routine HIV-1 drug resistance genotyping, would improve comparability across studies and support quality assurance.

#### 4.2.3 Statistical analysis

We recommend performing statistical comparisons of outcomes across HIV-1 subtypes using models that allow adjusting for relevant confounders. Appropriate methods should be used depending on study design, such as multivariate linear regression for cross-sectional analyses and mixed-effects models for longitudinal data. The chosen statistical approach, including model specifications, outcomes, and corresponding confidence intervals, should be clearly reported.

#### 4.2.4 Reporting and data availability

We urgently recommend standardized and transparent reporting of HIV-1 subtype classification, reservoir outcomes, and key participant data to enable cross-study comparison. Specifically, HIV-1 subtypes should be classified according to Los Alamos guidelines (42). In our dataset, subtypes were not reported in 112 of 263 articles describing the quantification of the HIV-1 reservoir, despite a search strategy specifically designed to identify such studies. This aligns with a previous systematic review, which reported that subtype information was provided in only 13% of studies (84). Even when subtypes were reported, the classification approach was often insufficiently described, particularly for recombinant forms. Adoption of standardized subtype definitions in line with Los Alamos guidelines, together with transparent reporting of subtype-specific results, would improve comparability and reproducibility across studies.

More detailed reporting of outcome data alongside individual participant metadata would improve interpretability and facilitate inclusion in meta-analyses. Providing individual-level data is urgently recommended, as it allows more robust adjustment for confounders. Where this is not feasible, studies should report summary statistics stratified by subtype, including appropriate measures of uncertainty.

We also urgently recommend systematically collecting and reporting key participant characteristics and timepoint-specific variables at the time of sampling that are known or hypothesized to influence the HIV-1 reservoir. At a minimum, studies should report confounders related to ART status and plasma viral load or suppression status. For treatment-naïve individuals or those at treatment initiation, the disease stage or duration of infection (or a proxy such as CD4 count) should be documented. For individuals on ART, the current ART regimen, treatment duration, duration of viral suppression, and the disease stage at treatment initiation should be reported (6, 15, 81). Furthermore, we recommend that studies report on key participant characteristics, such as geographic origin or ethnicity, biological sex, age, and co-infections, as these factors increasingly appear to influence the HIV-1 reservoir, although with less consistent conclusions (6, 21–23). Although additional host genetic factors like HLA type and CCR5 polymorphisms can also be considered. Importantly, women, children, and people with HIV-1 in non-Western settings were underrepresented in the included studies, underscoring the need for future research to assess the generalizability of current findings across these populations.

### 4.3 Strengths and limitations of our study

To the best of our knowledge, this is the first systematic review of studies quantifying the HIV-1 reservoir in peripheral blood across multiple non-B subtypes. However, several limitations should be considered. First, our analysis was restricted to peripheral blood, and future work should incorporate other anatomical compartments as well as indirect correlates of reservoir size or dynamics. For instance, integration site profiling could provide insights into reservoir dynamics and the maintenance or loss of virological control (26, 85, 86). Second, relevant studies may have been missed due to limitations of search terms; subtype-related information is not always consistently indexed in fields in PubMed and EMBASE, as illustrated by the three articles identified through reference screening. Moreover, publication bias cannot be excluded, as studies reporting no subtype-associated differences in HIV-1 reservoir size may have been underrepresented. Finally, heterogeneity across study methods and populations complicates cross-study comparisons.

### 4.4 Conclusion

In conclusion, this systematic review provides a comprehensive overview of studies measuring HIV-1 reservoir size in individuals with HIV-1 non-B subtypes and compiles a repository of reservoir outcomes with associated metadata. While findings were not entirely consistent, the available data suggest differences in reservoir size between subtype B and non-B subtypes, warranting further investigation. We also provide practical recommendations to strengthen study design, reporting, and comparability in future research, with a central recommendation being the routine consideration of HIV-1 subtypes. Together, these efforts advance our understanding of subtype-specific HIV-1 reservoir dynamics and support the development of globally effective HIV-1 cure strategies.

## Data Availability

The original contributions presented in the study are included in the article/**Supplementary Material**. Further inquiries can be directed to the corresponding authors.
