# Supplementary material for: Reservoir differences across HIV-1 subtypes: a systematic review to guide cure research
Source: Front Immunol. 2026 Jul 29;17:1870118. doi: 10.3389/fimmu.2026.1870118 (PMC13461633; doi:10.3389/fimmu.2026.1870118)
Supplement: Supplementary file 1 [file DataSheet1.docx]

Supplementary Material

# 1 Supplementary Data

## 1.1 Appendix 1: Search terms

MEDLINE: (("HIV"[Mesh:NoExp]) OR ( "HIV-1"[mh]) OR ("HIV-1"[tiab] ) OR ("Human Immunodeficiency Virus-1"[tiab] ) OR ("Human Immunodeficiency Virus"[ti] ) OR (hiv[ti] )) AND (("Genotype"[mh] ) OR ("Genotyping Techniques"[mh] ) OR ("Genetic Variation"[mh]) OR ("Serogroup"[mh]) OR (“subtyp*”[tiab]) OR ("intersubtyp*"[tiab]) OR ("intrasubtyp*"[tiab]) OR ("subsubtyp*"[tiab]) OR ( “clade”[ti] ) OR (“strain*”[ti] ) OR (“variant*”[ti] ) OR (“non-b”[tiab] ) OR (“non b ”[tiab] ) OR ("genotyp*"[tiab]) OR ("recombinant*"[ti]) OR ("unique recombinant form" [tiab] OR "URF"[tiab]) OR ("circulating recombinant form" [tiab] OR "CRF"[tiab]) OR ("genetic divers*"[tiab]) OR ("genetic varia*"[tiab]) OR ("serotyp*"[tiab])) AND (("Disease reservoirs/virology"[mh]) OR ("Virus latency"[mh]) OR ("Virus Activation"[mh]) OR ("reservoir*"[tiab]) OR ("HIV-1 DNA"[tiab]) OR ("HIV DNA"[tiab]) OR (“HIV-1 *viral DNA”[tiab]) OR ("laten*"[tiab] )) NOT ("Animals"[mh] NOT "Humans"[mh]) NOT (Case Reports[mh] OR (case-report*[tiab] OR case-stud*[tiab] OR case-ser*[tiab])) NOT (news[pt] OR congress[pt] OR book*[pt] )

EMBASE: (('human immunodeficiency virus'/de) OR ('human immunodeficiency virus 1'/exp) OR ('HIV-1':ab,ti) OR ('Human Immunodeficiency Virus-1':ab,ti) OR ('Human Immunodeficiency Virus':ti) OR ('hiv':ti)) AND (('genotype'/exp) OR ('genotyping'/exp) OR ('genetic variation'/exp) OR ('serotype'/exp) OR ('type strain'/exp) OR ('subtyp*':ti,ab,kw) OR ('intersubtyp*':ti,ab,kw) OR ('intrasubtyp*':ti,ab,kw) OR ('subsubtyp*':ti,ab,kw) OR ('clade':ti) OR ('strain*':ti) OR ('variant*':ti) OR ('non-b':ti,ab,kw) OR ('non b':ti,ab,kw) OR ('genotyp*':ti,ab,kw) OR ('recombinant*':ti) OR ('unique recombinant form':ti,ab,kw) OR (urf:ti,ab,kw) OR ('circulating recombinant form':ti,ab,kw) OR ('crf':ti,ab,kw) OR ('genetic divers*':ti,ab,kw) OR ('genetic varia*':ti,ab,kw) OR ('serotyp*':ti,ab,kw)) AND (('disease reservoir'/exp) OR ('virus latency'/exp) OR ('reservoir*':ti,ab,kw) OR ('hiv-1 dna':ti,ab,kw) OR ('hiv dna':ti,ab,kw) OR ('hiv-1 viral dna':ti,ab,kw) OR ('laten*':ti,ab,kw)) NOT ('case report'/de OR 'case study'/de OR (case-report* OR case-stud* OR case-ser*):ab,ti) NOT ([conference abstract]/lim) NOT ([animals]/lim NOT [humans]/lim)

## 1.2 Appendix 2: SPIRAL group authorship co-applicants

- Prof. Dr. Monique Nijhuis – UMC Utrecht; University of the Witwatersrand, Johannesburg, South Africa
- Dr. Godelieve de Bree – Amsterdam UMC
- Dr. Sarah E. Stutterheim – Maastricht University
- Prof. Dr. Tokameh Mahmoudi – Erasmus MC
- Dr. Alexander Pasternak – Amsterdam UMC
- Dr. Casper Rokx – Erasmus MC
- Dr. Cynthia Lungu – University of Zambia, Lusaka, Zambia
- Prof. Dr. Damalie Nakanjako – Makerere University College of Health Sciences, Kampala, Uganda
- Dr. David van de Vijver – Erasmus MC
- Dr. Elena Herrera Carrillo – CSIC, Granada, Spain
- Dr. Eva Vernooij – Utrecht University
- Dr. Ganna Rozhnova – UMC Utrecht
- Dr. Giulia Sinatti – Vrije Universiteit Amsterdam
- Dr. Jan A.C. Hontelez – Erasmus MC
- Dr. Jeroen den Dunnen – Amsterdam UMC
- Dr. Jori Symons – UMC Utrecht
- Dr. Kim Romijnders – UMC Utrecht
- Prof. Dr. Maria Papathanasopoulos – University of the Witwatersrand, Johannesburg, South Africa
- Dr. Marit van Gils – Amsterdam UMC
- Dr. Neeltje Kootstra – Amsterdam UMC
- Dr. Rob Gruters – Erasmus MC
- Dr. Rogier Sanders – Amsterdam UMC
- Dr. Shringar Rao – Erasmus MC
- Dr. Sody Munsaka – University of Zambia, Lusaka, Zambia
- Dr. Steven de Taeye – Amsterdam UMC
- Prof. Dr. Theo Geijtenbeek – Amsterdam UMC
- Dr. Thibault Mesplede – Erasmus MC
- Dr. Thumbi Ndung’u – Africa Health Research Institute, Durban, South Africa
- Dr. Yvonne M. Mueller – Erasmus MC

# 2 Supplementary Figures and Tables

## 2.1 Supplementary Figures

**Supplementary Figure 1: Suppression duration and ART initiation. (A)** Duration of viral suppression among participants on ART. Five studies included only participants not on ART, for two studies, treatment status was unknown. **(B)** Disease stages at treatment initiation across 33 studies. Study-defined classifications were used where available; otherwise, we defined acute infection as Fiebig I–II, early infection as <6 months post-infection, and chronic infection as >6 months post-infection.

**Supplementary Figure 2: Regions of the HIV-1 genome targeted for subtyping across included studies.** Numbers indicate the count of studies that targeted each genomic region. Studies may contribute to multiple regions if more than one target was used, as displayed by a dashed line.

**Supplementary Figure 3: Integrated, intact, and inducible measurements** (**A, B, D**) in individuals receiving ART, and (**B, C, E**) in individuals not on ART or with an unknown ART status, stratified by study, time point, and subtype. Boxplots are coloured by HIV-1 subtype and represent reservoir outcomes (interquartile range (IQR), median, mean, and/or minimum-maximum range, depending on the availability of summary statistics), for subtype groups with 3 or more samples. Boxplots are overlaid with individual data points when available. Reservoir quantifications are separated in subplots based on the reported units.

Brackets (“]”) indicate statistical comparisons by the authors of the included study (* p < 0.05; ** p<0.005; “n.s”, non-significant). “Other” denotes all remaining subtypes and those not explicitly reported in a given study. Data for unknown subtypes is not shown. For studies that performed multiple assays on the same sample, one assay was shown.

## 2.2 Supplementary Tables

**Table S1:** PRISMA 2020 checklist for systematic reviews. (Page, McKenzie, et al., 2021)

| **Section and Topic** | **Item #** | **Checklist item** | **Location where item is reported** |
| --- | --- | --- | --- |
| **TITLE** | | |  |
| Title | 1 | Identify the report as a systematic review. | Frontpage |
| **ABSTRACT** | | |  |
| Abstract | 2 | See the PRISMA 2020 for Abstracts checklist. | Abstract |
| **INTRODUCTION** | | |  |
| Rationale | 3 | Describe the rationale for the review in the context of existing knowledge. | Section 1 |
| Objectives | 4 | Provide an explicit statement of the objective(s) or question(s) the review addresses. | Section 1 |
| **METHODS** | | |  |
| Eligibility criteria | 5 | Specify the inclusion and exclusion criteria for the review and how studies were grouped for the syntheses. | Section 2.2 |
| Information sources | 6 | Specify all databases, registers, websites, organisations, reference lists and other sources searched or consulted to identify studies. Specify the date when each source was last searched or consulted. | Section 2.1 |
| Search strategy | 7 | Present the full search strategies for all databases, registers and websites, including any filters and limits used. | Appendix 1 |
| Selection process | 8 | Specify the methods used to decide whether a study met the inclusion criteria of the review, including how many reviewers screened each record and each report retrieved, whether they worked independently, and if applicable, details of automation tools used in the process. | Section 2.2 |
| Data collection process | 9 | Specify the methods used to collect data from reports, including how many reviewers collected data from each report, whether they worked independently, any processes for obtaining or confirming data from study investigators, and if applicable, details of automation tools used in the process. | Section 2.3 |
| Data items | 10a | List and define all outcomes for which data were sought. Specify whether all results that were compatible with each outcome domain in each study were sought (e.g. for all measures, time points, analyses), and if not, the methods used to decide which results to collect. | Section 2.3 |
|  | 10b | List and define all other variables for which data were sought (e.g. participant and intervention characteristics, funding sources). Describe any assumptions made about any missing or unclear information. | Section 2.3 |
| Study risk of bias assessment | 11 | Specify the methods used to assess risk of bias in the included studies, including details of the tool(s) used, how many reviewers assessed each study and whether they worked independently, and if applicable, details of automation tools used in the process. | Section 2.4 |
| Effect measures | 12 | Specify for each outcome the effect measure(s) (e.g. risk ratio, mean difference) used in the synthesis or presentation of results. | Section 2.5 |
| Synthesis methods | 13a | Describe the processes used to decide which studies were eligible for each synthesis (e.g. tabulating the study intervention characteristics and comparing against the planned groups for each synthesis (item #5)). | Section 2.5 |
|  | 13b | Describe any methods required to prepare the data for presentation or synthesis, such as handling of missing summary statistics, or data conversions. | Section 2.5 |
|  | 13c | Describe any methods used to tabulate or visually display results of individual studies and syntheses. | Section 2.5 |
|  | 13d | Describe any methods used to synthesize results and provide a rationale for the choice(s). If meta-analysis was performed, describe the model(s), method(s) to identify the presence and extent of statistical heterogeneity, and software package(s) used. | Section 2.5 |
|  | 13e | Describe any methods used to explore possible causes of heterogeneity among study results (e.g. subgroup analysis, meta-regression). | NA |
|  | 13f | Describe any sensitivity analyses conducted to assess robustness of the synthesized results. | NA |
| Reporting bias assessment | 14 | Describe any methods used to assess risk of bias due to missing results in a synthesis (arising from reporting biases). | NA |
| Certainty assessment | 15 | Describe any methods used to assess certainty (or confidence) in the body of evidence for an outcome. | NA |
| **RESULTS** | | |  |
| Study selection | 16a | Describe the results of the search and selection process, from the number of records identified in the search to the number of studies included in the review, ideally using a flow diagram. | Section 3.1, Figure 1 |
|  | 16b | Cite studies that might appear to meet the inclusion criteria, but which were excluded, and explain why they were excluded. | Section 3.1 |
| Study characteristics | 17 | Cite each included study and present its characteristics. | Table 2 |
| Risk of bias in studies | 18 | Present assessments of risk of bias for each included study. | Section 3.2, Figure 2, Table S2 |
| Results of individual studies | 19 | For all outcomes, present, for each study: (a) summary statistics for each group (where appropriate) and (b) an effect estimate and its precision (e.g. confidence/credible interval), ideally using structured tables or plots. | Section 3.6 - 3.11, Table 1, 2, S2, Figure 4-7, S1-S3 |
| Results of syntheses | 20a | For each synthesis, briefly summarise the characteristics and risk of bias among contributing studies. | Figure 2-4, 7 |
|  | 20b | Present results of all statistical syntheses conducted. If meta-analysis was done, present for each the summary estimate and its precision (e.g. confidence/credible interval) and measures of statistical heterogeneity. If comparing groups, describe the direction of the effect. | Figure 7 |
|  | 20c | Present results of all investigations of possible causes of heterogeneity among study results. | Section 3.3 - 3.5, Figure 2, 3 |
|  | 20d | Present results of all sensitivity analyses conducted to assess the robustness of the synthesized results. | NA |
| Reporting biases | 21 | Present assessments of risk of bias due to missing results (arising from reporting biases) for each synthesis assessed. | NA |
| Certainty of evidence | 22 | Present assessments of certainty (or confidence) in the body of evidence for each outcome assessed. | NA |
| **DISCUSSION** | | |  |
| Discussion | 23a | Provide a general interpretation of the results in the context of other evidence. | Section 4.1 |
|  | 23b | Discuss any limitations of the evidence included in the review. | Section 4.2 |
|  | 23c | Discuss any limitations of the review processes used. | Section 4.2, 4.3 |
|  | 23d | Discuss implications of the results for practice, policy, and future research. | Section 4.2.1-4.2.4, 4.4 |
| **OTHER INFORMATION** | | |  |
| Registration and protocol | 24a | Provide registration information for the review, including register name and registration number, or state that the review was not registered. | Section 2.1 |
|  | 24b | Indicate where the review protocol can be accessed, or state that a protocol was not prepared. | Section 2.1 |
|  | 24c | Describe and explain any amendments to information provided at registration or in the protocol. | NA |
| Support | 25 | Describe sources of financial or non-financial support for the review, and the role of the funders or sponsors in the review. | Section 7 |
| Competing interests | 26 | Declare any competing interests of review authors. | Section 5 |
| Availability of data, code and other materials | 27 | Report which of the following are publicly available and where they can be found: template data collection forms; data extracted from included studies; data used for all analyses; analytic code; any other materials used in the review. | Section 10 |

**Table S2:** A data sheet with all included studies and individual data is made available as a separate Excel file.
